# Schwann cell autophagy induced by SAHA, 17-AAG, or clonazepam can reduce bortezomib-induced peripheral neuropathy

**DOI:** 10.1038/sj.bjc.6605954

**Published:** 2010-10-19

**Authors:** T Watanabe, K Nagase, M Chosa, K Tobinai

**Affiliations:** 1 Hematology Division, National Cancer Center Hospital, 5-1-1 Tsukiji, Chuo-ku, Tokyo 104-0045, Japan

**Keywords:** autophagy, multiple myeloma, neuropathy, proteasome inhibitors

## Abstract

**Background:**

The proteasome inhibitor bortezomib has improved the survival of patients with multiple myeloma but bortezomib-induced peripheral neuropathy (BiPN) has emerged as a serious potential complication of this therapy. Animal studies suggest that bortezomib predominantly causes pathological changes in Schwann cells. A tractable system to evaluate combination drugs for use with bortezomib is essential to enable continuing clinical benefit from this drug.

**Methods:**

Rat schwannoma cells were pretreated with vincristine (VCR), histone deacetylase inhibitors, anticonvulsants, or a heat-shock protein 90 (HSP90) inhibitor. To then monitor aggresome formation as a result of proteasome inhibition and the activation of chaperone-mediated autophagy (CMA), we performed double-labelling immunofluorescent analyses of a cellular aggregation-prone protein marker.

**Results:**

Aggresome formation was interrupted by VCR, whereas combination treatments with bortezomib involving suberoylanilide hydroxamic acid, 17-allylamino-17-demethoxy-geldanamycin, or clonazepam appear to facilitate the disposal of unfolded proteins via CMA, inducing HSP70 and lysosome-associated membrane protein type 2A (LAMP-2A).

**Conclusions:**

This schwannoma model can be used to test BiPN-reducing drugs. The present data suggest that aggresome formation in Schwann cells is a possible mechanism of BiPN, and drugs that induce HSP70 or LAMP-2A have the potential to alleviate this complication. Combination clinical trials are warranted to confirm the relevance of these observations.

The development of novel agents, such as proteasome inhibitors and immunomodulatory drugs has improved the survival outcome for multiple myeloma (MM) patients (Kumar *et al*, 2008). However, the incidence of peripheral neuropathy (PN) has emerged as a significant problem in the new therapeutic era for MM (Richardson *et al*, 2006, 2009b; Argyriou *et al*, 2008). In younger patients with MM, primary treatments have included vincristine (VCR), doxorubicin, and dexamethasone, and also high-dose therapy with melphalan supported by autologous stem cell transplantation. However, in some of these patients, VCR treatments have caused PN. Moreover, bortezomib was the first proteasome inhibitor to be approved for the treatment of relapsed/refractory as well as newly diagnosed MM patients (Richardson *et al*, 2003; San Miguel *et al*, 2008). However, this treatment can cause peripheral nerve damage leading to the development of bortezomib-induced peripheral neuropathy (BiPN). Owing to these adverse effect, bortezomib will be discontinued even in patients that respond well to this drug. Not surprisingly, bortezomib has recently become one of the mainstays in ongoing clinical trials of combination therapies for MM.

A couple of recent studies have reported neurophysiological and pathological findings for bortezomib administration in animal models (Cavaletti *et al*, 2007; Bruna *et al*, 2010; Meregalli *et al*, 2010). Another histopathological study in rats reported that bortezomib did not affect neurons but did cause damage to Schwann cells (Cavaletti *et al*, 2007). Another report has however shown that alterations to Schwann cells might be a secondary effect of bortezomib (Bruna *et al*, 2010). At present, treatments for BiPN are lacking, although anticonvulsants have been administered to MM patients with this disorder (Richardson *et al*, 2006; Argyriou *et al*, 2008). In addition, although a dose-modification guideline for BiPN has been published (Richardson *et al*, 2009b), it is difficult to accurately evaluate neurotoxicity in patients during bortezomib therapy and thus determine when treatment should discontinue. Hence, combination bortezomib treatments for MM involving agents that function as prophylactics against BiPN, rather than drugs that treat BiPN, are highly desirable. However, there are currently few (if any) investigative tools available to develop such therapies as the molecular mechanisms underlying BiPN remain to be elucidated.

To elucidate the molecular mechanisms underpinning the onset of BiPN in our current study, we first reviewed previous reports on neurodegenerative diseases in which protein aggregates are responsible for the cellular toxicity. When the activity of proteasome is inhibited, misfolded proteins will form aggregates known as aggresomes (Johnston *et al*, 1998). Aggresomes were initially described as inclusion bodies in the cells of patients with neurodegenerative diseases (Kopito, 2000) such as amyotrophic lateral sclerosis (Bruijn *et al*, 1998; Mezey *et al*, 1998), Parkinson's disease (Mezey *et al*, 1998), and Huntington's disease (Bennett *et al*, 2007). In our present experiments, we employed a schwannoma cell system to monitor aggresome formation after treatment with bortezomib. Furthermore, we examined whether additional treatments could reduce the number and size of these aggregates and thus potentially suppress the onset of BiPN.

## Materials and methods

### Schwann cell pretreatment and bortezomib treatment

A rat schwannoma cell line RT4-D6P2T (purchased from ATCC, Manassas, VA, USA, on 28 May 2007) was cultured in Dulbecco's modified Eagle's medium (Sigma-Aldrich, St Louis, MO, USA) containing 10% FBS (Bioserum, Victoria, Australia). RT4-D6P2T cells were cultured for less than 2 months after reconstitution from stocks, which were frozen upon receipt from the ATCC. The cells had been validated by the supplier using DNA fingerprinting and no additional authentication was performed in our laboratory. The morphology of the RT4-D6P2T cells showed no changes over the course of the study.

At 1 day before pretreatment, the RT4-D6PT2 cells were plated at a density of 5 × 10^4^ cells per well on four-well chamber slides. They were then either untreated or pretreated with 40 nM VCR (Sigma-Aldrich) for 1 h or pretreated for 24 h with either 5 *μ*M suberoylanilide hydroxamic acid (SAHA; Merck & Co. Inc., Whitehouse Station, NJ, USA), 0.5 *μ*M 17-allylamino-17-demethoxy-geldanamycin (17-AAG; Sigma-Aldrich), 50 nM clonazepam (CZP; Sigma-Aldrich), or 6 mM valproic acid (VPA; Sigma-Aldrich). The dose of each reagent was determined by its half maximal inhibitory value (IC_50_). For VCR pretreatments, the cells were washed twice with PBS: 2.68 mM KCl, 1.47 mM KH_2_PO_4_, 136.89 mM NaCl, and 8.10 mM Na_2_HPO_4_ (Dainippon Sumitomo Pharma Co. Ltd., Osaka, Japan) before the addition of 40 nM bortezomib (Millennium Pharmaceuticals, Cambridge, MA, USA) for 3 h. Following pretreatment with other reagents, the cells were not washed before the 3-h treatment with 40 nM bortezomib. As a final step, the cells were washed twice with PBS, incubated for a further 24 h, and then fixed.

### Immunohistochemical analysis

The RT4-D6P2T cells were fixed with PBS containing 4% paraformaldehyde for 10 min at 4°C, washed with TBS (20 mM Tris and 500 mM NaCl (pH 7.4)) with 0.1% IGEPAL CA-630 (Fluka, Buchs, Switzerland) for 3 × 5 min, fixed in methanol for 10 min at 4°C, and blocked with PBS containing 4% BSA (Sigma-Aldrich) for 30 min at room temperature. The cells were then incubated overnight at 4°C with primary antibodies diluted at a ratio of 1 : 50 in PBS with 4% BSA (*γ*-tubulin (Sigma-Aldrich), dynein (Sigma-Aldrich), vimentin (Santa Cruz Biotechnology, Santa Cruz, CA, USA), heat-shock protein 70 (HSP70; Santa Cruz Biotechnology), peripheral myelin protein 22 (PMP22; Millipore, Bedford, MA, USA), and lysosome-associated membrane protein type 2A (LAMP-2A; Abcam, Cambridge, MA, USA)). The cells were then washed 3 × 5 min in TBS with 0.1% IGEPAL CA-630 and incubated with secondary antibodies diluted at a ratio of 1 : 100 in PBS with 4% BSA, for 1 h at room temperature (Alexa Fluor 488-conjugated chicken anti-rabbit IgG and Alexa Fluor 555-conjugated goat anti-mouse IgG (Molecular Probes, Eugene, OR, USA)). After a further washing for 3 × 5 min in TBS with 0.1% IGEPAL CA-630, the cells were mounted on slides with VECTASHIELD (Vector Laboratories, Burlingame, CA, USA). We note that all washes were performed at room temperature. Images of the cells were captured on a laser scanning confocal microscope BZ-8000 (Keyence, Osaka, Japan) and analysed by BZ-Analyser software (Keyence). The thickness of the optical sections analysed was 0.4 *μ*m.

### Quantification of aggresomes and round structures outside of the Schwann cells

Aggresomes and round structures outside of the cells were identified by the colocalisation of PMP22 and *γ*-tubulin, counted in triplicate from 200 cells, and expressed as a percentage of the total cells.

### Growth inhibition assay of MM cells

The human MM cell lines, MM.1S, RPMI8226 (purchased from ATCC), and KMS-18 (kindly provided by Dr T Otsuki, Department of Hygiene, Kawasaki Medical School, Kurashiki, Japan) were maintained in RPMI1640 (Sigma-Aldrich) containing 10% FBS. The growth-inhibitory effects upon MM cells were determined using a 3-(4, 5-dimethyl-2-thiazolyl)-2,5-diphenyl-2H-tetrazolium bromide (MTT) assay (Sigma-Aldrich). At 1 day before treatment, 9.0 × 10^4^ cells per 90 *μ*l aliquot were cultured in 96-well plates (Sumitomo Bakelite, Higashikangawa, Japan) in triplicate at 37°C. Cells were either untreated or pretreated for 24 h with the same concentration of each reagent used with the RT4-D6P2T cells except for VCR. The cells were then cultured further with varying concentrations (from 0.5 to 3 nM) of bortezomib for 48 h. Optical densities (ODs) at 570 and 630 nm were measured using a multiplate reader. Stock MTT was added to each of the wells in the assay, and the plates were further incubated at 37°C for 5 h. Dimethyl sulphoxide (Sigma-Aldrich) was added to all wells and mixed thoroughly. After a few minutes at room temperature to ensure that all formazan crystals were dissolved, the plates were read on a SpectroMax 340PC^384^ VersaMax (Molecular Devices, Sunnyvale, CA, USA), using a test wavelength of 570 nm and a reference wavelength of 630 nm. Cell growth (%) was calculated as follows: (OD_630_–OD_570_ of the samples/OD_630_−OD_570_ of the control) × 100.

## Results

### Aggresomes form at MTOC following proteasome inhibition in Schwann cells

**Figure 1:**
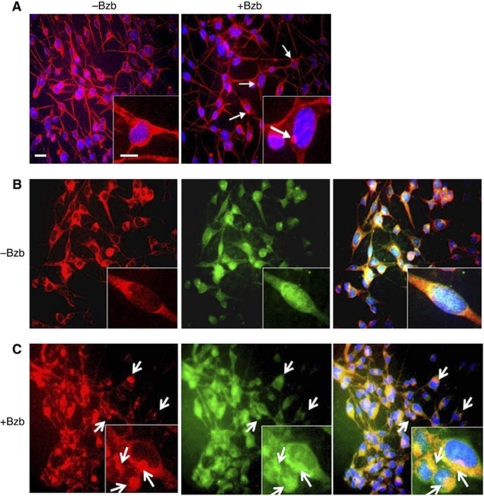
Bortezomib induces aggresome formation at the microtubule-organising centres (MTOCs) of Schwann cells. (**A**) In untreated RT4-D6P2T cells, *γ*-tubulin is distributed homogeneously throughout the cytoplasm (left panel). In bortezomib (Bzb)-treated cells, aggresomes form as distinct pericentriolar structures (arrows) with weak staining in the cytoplasm (right panel). Insets in the right panel show the juxtanuclear rounded structures evident at higher magnification. (**B**) Untreated RT4-D6P2T cells contain dynein (red), which is distributed homogeneously in the cytoplasm with predominant localisation in the perinuclear region, and vimentin (green), which is distributed diffusely throughout the cytoplasm and above the nuclei. (**C**) In bortezomib-treated cells, dynein (red) and vimentin (green) appear as rounded structures at the MTOC (arrows) and are colocalised in the region adjacent to the nuclei (yellow signals in the merged image of both fluorochrome channels. Bar, 20 *μ*M. −Bzb, untreated; +Bzb, bortezomib treated.

A diffuse expression pattern of *γ*-tubulin, a protein that adheres to the centrosome (Dictenberg *et al*, 1998), was observed in the cytoplasm of RT4-D6P2T cells. Following a 3-h treatment with 40 nM bortezomib, however, *γ*-tubulin staining in the cytoplasm became weak and coalesced to form round structures in the juxtanuclear area (Figure 1A). Similarly, the dynein and vimentin proteins became rounded and colocalised in region adjacent to the nucleus after exposure to bortezomib (Figure 1C).

### Vincristine abrogates bortezomib-induced aggresome formation and combination treatments augment the exocytosis of endogenous misfolded proteins from Schwann cells

**Figure 2:**
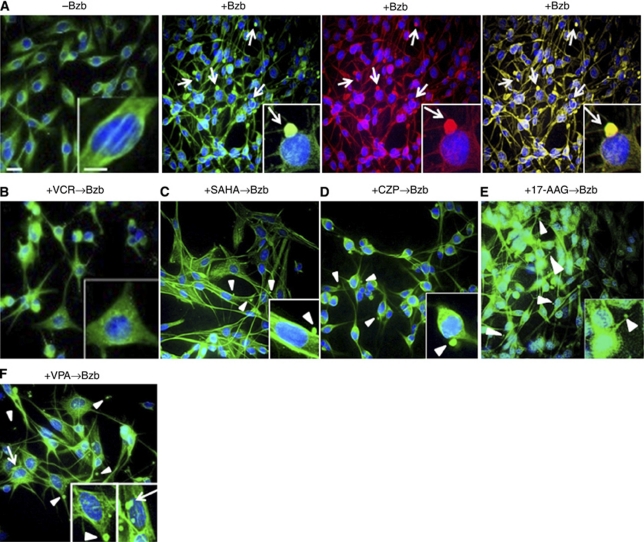
Vincristine (VCR) abrogates aggresome formation and in a combination treatment with bortezomib augments the exocytosis of endogenous misfolded proteins. (**A**) Peripheral myelin protein 22 (PMP22) is homogeneously distributed throughout the cytoplasm of RT4-D6P2T cells before treatment with bortezomib (left panel). After treatment with bortezomib (Bzb), PMP22 appears to undergo retrograde transport towards the MTOC where it forms perinuclear aggresomes (arrows, middle panel, green signals) and colocalises with *γ*-tubulin (arrows, middle panel, red signals). A merged image of both fluorophores is shown in the far right panel (yellow signal). (**B**) Following pretreatment with VCR, a microtubule depolymerisation agent, PMP22 signals are evident at multiple sites in a granular pattern of aggregates throughout the cytoplasm, most notably in the perikaryon. Cells pretreated with (**C**) suberoylanilide hydroxamic acid (SAHA), a known histone deacetylase inhibitor (HDACi), or (**D**) clonazepam (CZP), an anticonvulsant, and (**E**) 17-allylamino-17-demethoxy-geldanamycin (17-AAG), a HSP90 inhibitor, fail to form aggresomes, but instead form rounded structures outside of the cell (arrowheads), which are smaller than the perinuclear aggresomes. (**F**) Pretreatment with valproic acid (VPA) causes the appearance of similar rounded structures outside of the cells (arrowheads) in addition to juxtanuclear aggresomes (arrows).

We next examined whether endogenous misfolded proteins destined to be processed by the ubiquitin-proteasome system could be induced to aggregate and undergo retrograde transport towards the microtubule-organising center (MTOC) upon proteasome inhibition. To accomplish this, we employed the cellular marker PMP22, a short-lived glycoprotein present in Schwann cells (Fortun *et al*, 2003). Following bortezomib treatment, PMP22 showed a distinct juxtanuclear and rounded appearance and colocalised with *γ*-tubulin to form aggresomes (Figure 2A), as previously reported (Fortun *et al*, 2003). Interestingly, treatments with VCR completely abrogated the bortezomib-induced accumulation of PMP22, which was instead observed as numerous spots in the perikaryon (Figure 2B).

**Figure 3:**
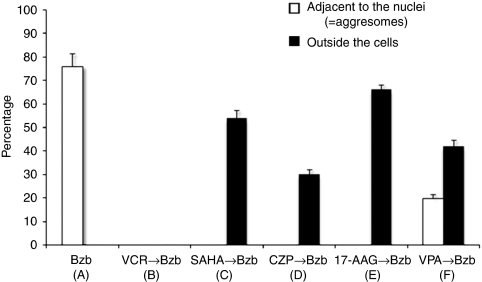
The percentages of round structures adjacent to the nuclei (i.e., aggresomes) and outside the cells were calculated from images showing the colocalisation of PMP22 and *γ*-tubulin (yellow signals in the far right panel in Figure 2A). These numbers were measured in triplicate and are expressed as the means±s.d. The letters in parentheses under the treatment categories correspond to the images above.

We next analysed whether treatments with a combination of reagents could reduce aggresome formation. Intriguingly, pretreatment with the histone deacetylase inhibitor (HDACi) SAHA (Figure 2C), the anticonvulsant CZP (Figure 2D), or the HSP90 inhibitor 17-AAG (Figure 2E) caused the appearance of round structures, which were smaller than aggresomes, outside of the cells and with no juxtanuclear aggresomes (Figure 3). In contrast, pretreatment with VPA, also an anticonvulsant and an HDACi, caused the appearance of rounded structures outside of the cells in addition to juxtanuclear aggresomes (Figures 2F and 3).

### Chaperone-mediated autophagy is responsible for the enhanced exocytosis of misfolded proteins in Schwann cells during proteasome inhibition

**Figure 4:**
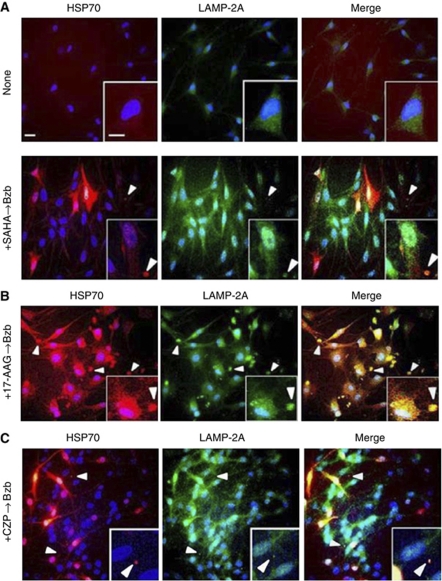
Combination treatments with bortezomib can augment the exocytosis of misfolded proteins through the chaperone-mediated autophagy of Schwann cells. The distributions of HSP70/HSC70 (red), a chaperone protein, and LAMP-2A (green), a lysosomal membrane protein with a specific role in chaperone-mediated autophagy, are shown in response to combination treatments with (**A**) SAHA, (**B**) 17-AAG, and (**C**) CZP. The colocalisation of both proteins is evidenced by the small rounded structures outside of the cells that appear as an orange signal (arrowheads). Bar, 20 *μ*M.

To analyse the molecular mechanisms underlying the enhanced exocytosis of misfolded proteins in Schwann cells, we used an antibodies against the HSP70 chaperone protein and the receptor for chaperone-mediated autophagy (CMA) at the lysosomal membrane (which is a unique isoform of LAMP-2, LAMP-2A) (Cuervo and Dice, 2000; Kaushik *et al*, 2006). After treatment with SAHA, 17-AAG, or CZP (Figure 4A, B, or C, respectively) followed by bortezomib, HSP70 and LAMP-2A were found to colocalise in structures outside of the cells.

### Drugs that protect Schwann cells from aggresome formation due to bortezomib treatment do not disrupt the growth inhibitory effects of bortezomib in myeloma cells

**Figure 5:**
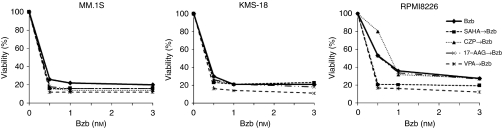
The same combinations used in Figures 2 and 3 do not suppress the growth inhibition of multiple myeloma (MM) cells induced by bortezomib. In MM, cells (MM.1S, KMS-18, and RPMI8226) were treated with bortezomib alone or in combination with SAHA, CZP, 17-AAG, or VPA. The proportion of viable cells after pretreatment with each drug followed by bortezomib treatment is indicated as a percentage of the untreated cells. These numbers were measured in triplicate and are expressed as the means±s.d.

Pretreatments of MM cells with the same drugs used in the RT4-D6P2T cell experiments had few negative effects on the profound growth inhibitory effects of bortezomib (Figure 5).

## Discussion

The findings of our present study using a schwannoma cell model system suggest that aggresome formations caused by proteasome inhibition and the excretion pathways of intracellular misfolded proteins are targets for combination drug candidates that will alleviate the onset of BiPN during bortezomib treatment.

A recent study of skin biopsies has revealed that BiPN manifests as predominantly large fibres (Chaudhry *et al*, 2008). On the other hand, in some BiPN patients who develop treatment-emergent neuropathy, the underlying cause has been attributed to the impairment of small fibres (Richardson *et al*, 2006), even though such fibres comprise myelinated A*δ* and unmyelinated C fibres. In contrast, it has been proposed that 68–85% of BiPN cases are reversible (Richardson *et al*, 2009a, 2009b).

Although it has already been demonstrated that the behaviour of cells of neoplastic origin can differ markedly from normal cells (Scuteri *et al*, 2006), cell lines are usually more tractable for experimental purpose than primary culture cells. In addition, because BiPN is predominantly sensory (Richardson *et al*, 2006; Richardson *et al*, 2009a, 2009b), it would have been desirable to use cell lines that would somewhat mimic the peripheral sensory nerves. No such cells are currently available however and we thus employed schwannoma cells for analysis, which are benign and differentiated tumour cells, rather than neuroblastoma cells used in previous reports (Scuteri *et al*, 2006; Csizmadia *et al*, 2008).

Our present data are consistent with previous observations that misfolded proteins form aggregates throughout the cell if they are not degraded by the proteasome (Figure 2B). Furthermore, such aggregates are then transported in a microtubule (MT)-dependent manner to the MTOC on the dynein motor complex (Figure 1C, red) (Johnston *et al*, 1998; Kopito, 2000; Garcia-Mata *et al*, 2002). After treatment with bortezomib, it has been shown that vimentin, the most common component of the intermediate filament cytoskeleton (Franke *et al*, 1978), collapses to form a ‘cage’ surrounding the aggresome, which then adopts a ‘rounded’ morphology (Figure 1C, green) (Johnston *et al*, 1998; García-Mata *et al*, 1999). Moreover, our observations of aggresome formations with a distinct juxtanuclear spherical appearance that colocalise with *γ*-tubulin (Figure 1A, right) after treatment with proteasome inhibitor in Schwann cells corroborate those of a previous study (Fortun *et al*, 2003). Moreover, our results demonstrating that the fate of intracellular ubiquitinated aggregation-prone proteins may be relevant to the development of BiPN support previous findings for the gene expression profiles of bone marrow cells in MM patients with treatment-emergent BiPN (Richardson *et al*, 2009b). These authors identified distinct classes of gene transcripts, namely those involved in the initiation and regulation of protein translation, and their results indicated that enriched proteins that are released from MM cells may be toxic to the peripheral nervous system (Richardson *et al*, 2009b).

**Figure 6:**
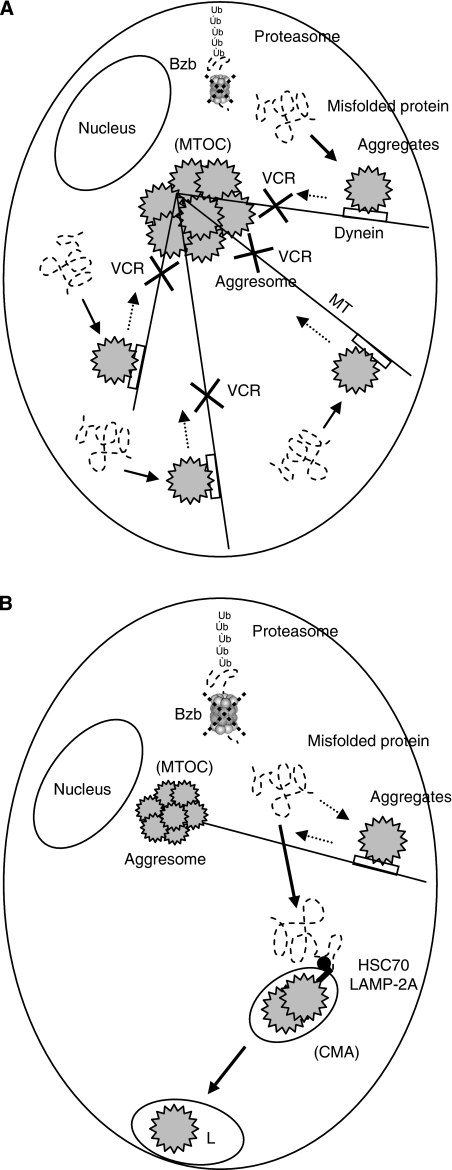
Schematic representation of the disruption of aggresome formation (**A**) and chaperone-mediated autophagy (CMA) (**B**). (**A**) Small peripherally formed aggregates are transported along the microtubule (MT) tracks by retrograde motors (i.e., dyneins) to a juxtanuclear pericentriolar location, the MT organisation centre (MTOC). ‘Xvcr’ indicates that VCR pretreatment before the administration of bortezomib (Bzb) hinders aggresome formation. This is likely because VCR is an MT-disrupting drug and the aggregates would be unable to move along the MTs on the dyneins towards the MTOC. (**B**) SAHA, CZP, 17-AAG, and VPA have the potential to enhance the expression of HSP70/heat-shock cognate protein of 70 Kd (HSC70), which recognises the specific motif targeted by CMA in its substrate proteins. Lysosome-associated membrane protein type 2A (LAMP-2A) is a unique receptor for CMA. Aggregated proteins are delivered from the cytoplasm out of the cells through lysosomes (L) by CMA.

PMP22 is associated with a demyelinating PN, Charcot–Marie–Tooth disease type 1A (Patel *et al*, 1992), and VCR is contraindicated in patients with this disease. In our present study, we observed that VCR treatment resulted in the dispersion of aggregates in the cytoplasm and no formation of juxtanuclear aggresomes (Figure 2B). In other words, because VCR is an MT-disrupting drug, our result suggests that pretreatment with this agent might increase BiPN by hindering the movement of unfolded proteins along the MTs with dynein motor complexes (Figure 6A). Indeed, other investigators have suggested that the neuropathy produced by VCR treatment may compromise the ability of the patients to receive bortezomib (Kyle and Rajkumar, 2009).

The central aim of our current study was to develop a clinically relevant *in vitro* system to test drugs that could be combined with bortezomib to reduce the incidence of BiPN. One of the tested candidates was the anticonvulsant VPA, which has been used previously to alleviate the symptoms of painful diabetic neuropathy (Kochar *et al*, 2004). However, the 6 mM concentration of VPA used in our experiments is more than 4000-fold higher than the previously reported clinical dosage (Munster *et al*, 2009). Furthermore, our results suggest that VPA may be less effective in reducing BiPN than other HDACi's such as SAHA or anticonvulsants like CZP. Indeed, pretreatment with VPA followed by bortezomib was found to elicit juxtanuclear aggresome formation in addition to the formation of rounded structures outside of the cells (Figures 2F and 3). On the other hand, SAHA has been shown previously to disrupt bortezomib-induced aggresome formation in MM cells (Nawrocki *et al*, 2008) as a result of the destruction of HDAC6, which promotes aggresome inclusion of misfolded polyubiquitylated proteins on the dynein motor complexes along the MTs (Kawaguchi *et al*, 2003). The 5 *μ*M concentration of SAHA used in this study was two- to five-fold higher than the clinically usable dose in our previous pharmacokinetic analyses of phase I trials of oral SAHA (Watanabe *et al*, 2010). However, the 40 nM quantity of bortezomib used in this study is equivalent to that observed in our earlier study (Ogawa *et al*, 2008), and the 40 nM of VCR, 50 nM of CZP, and 0.5 *μ*M of 17-AAG used in our analyses are equivalent to the doses for these compounds reported in other studies (Goetz *et al*, 2005; Corona *et al*, 2008; dos Santos *et al*, 2009, respectively).

The results of our current analyses shown in Figure 4 suggest that following pretreatment with the candidate drugs, the aggregated proteins are discarded outside of the cells by CMA (Kaushik *et al*, 2006). The evidence in support of CMA as the mechanism of disposal in this case is that the antibody used in our experiments does not distinguish between HSP70 and the heat-shock cognate protein of 70 Kd (HSC70) (Shen *et al*, 2009), which recognises the CMA-targeting motif in the substrate protein (Agarraberes *et al*, 1997). To our knowledge, the role of CMA either under conditions of proteasome inhibition or in the nervous system has never been previously reported. However, HSP70 and LAMP-2A, a specific receptor for CMA, were found in our analysis to be colocalised in the rounded structures including misfolded proteins (Figure 4). By inducing the chaperone protein, we speculate that these agents may promote an additional degradation pathway via lysosomes to excrete aggregated proteins from Schwann cells. This is different from the retrograde transport of aggregated proteins to form aggresomes along MTs from the periphery in the cytoplasm to the MTOC, thus aiding cells in the disposal of aggregated proteins (Figure 6B).

The overexpression of HSP70, which could be induced by SAHA alone in our experiments (Figure 4A, data not shown), is a well-described consequence of HSP90 inhibition by 17-AAG (Guo *et al*, 2005). This finding is consistent with the results from series of previous reports, which showed that a pan-HDACi similar to SAHA inhibits the HSP90 deacetylase HDAC6 (Bali *et al*, 2005), and that acetylation of HSP90 releases heat-shock factor-1 from HSP90 (Zou *et al*, 1998) and consequently induces HSP70 expression (Morimoto, 1998). Furthermore, our present *in vitro* data may corroborate the results of a clinical trial with bortezomib and tanespimycin (a cremophor-based formulation of 17-AAG) in which BiPN was reduced (Mitsiades *et al*, 2009; Richardson *et al*, 2010). In the case of SAHA, a multicentre phase I trial in combination with bortezomib for relapsed or refractory MM patients has been performed and only mild PN was reported (Badros *et al*, 2009). Another case series has reported gastrointestinal tract events only without discontinuation or dose adjustments of either agent (Mazumder *et al*, 2010). Interestingly, HSP70 has also been show to have a major role in the cellular defence against the toxic effects of misfolded proteins in neurodegenerative diseases such as amyotrophic lateral sclerosis (Gifondorwa *et al*, 2007), Parkinson's disease (Roodveldt *et al*, 2009), and Huntington's disease (Wacker *et al*, 2009).

As the binding of substrates, that is, misfolded proteins, to LAMP-2A is the limiting step for degradation via CMA (Cuervo and Dice, 1996), the induction of LAMP-2A as well as HSP70/HSC70 may be a promising marker for screening drugs that may reduce BIPN.

In summary, although the results of our present study are preliminary and *in vitro* only, our data suggest that the combination of bortezomib and SAHA, 17-AAG, or CZP has the potential to reduce BiPN. As bortezomib is currently an important component of combination treatment for MM, our *in vitro* system may allow MM patients to continue to benefit from bortezomib in the future.

## References

[bib1] Agarraberes FA, Terlecky SR, Dice JF (1997) An intralysosomal hsp70 is required for a selective pathway of lysosomal protein degradation. J Cell Biol 137: 825–834 9151685 10.1083/jcb.137.4.825 PMC2139836

[bib2] Argyriou AA, Iconomou G, Kalofonos HP (2008) Bortezomib-induced peripheral neuropathy in multiple myeloma: a comprehensive review of the literature. Blood 112: 1593–1599 18574024 10.1182/blood-2008-04-149385

[bib3] Badros A, Burger AM, Philip S, Niesvizky R, Kolla SS, Goloubeva O, Harris C, Zwiebel J, Wright JJ, Espinoza-Delgado I, Baer MR, Holleran JL, Egorin MJ, Grant S (2009) Phase I study of vorinostat in combination with bortezomib for relapsed and refractory multiple myeloma. Clin Cancer Res 15: 5250–5257 19671864 10.1158/1078-0432.CCR-08-2850 PMC2758911

[bib4] Bali P, Pranpat M, Bradner J, Balasis M, Fiskus W, Guo F, Rocha K, Kumaraswamy S, Boyapalle S, Atadja P, Seto E, Bhalla K (2005) Inhibition of histone deacetylase 6 acetylates and disrupts the chaperone function of heat shock protein 90: a novel basis for antileukemia activity of histone deacetylase inhibitors. J Biol Chem 280: 26729–26734 15937340 10.1074/jbc.C500186200

[bib5] Bennett EJ, Shaler TA, Woodman B, Ryu K-Y, Zaitseva TS, Becker CH, Bates GP, Schulman H, Kopito RR (2007) Global changes to the ubiquitin system in Huntington's disease. Nature 448: 704–708 17687326 10.1038/nature06022

[bib6] Bruijn LI, Houseweart MK, Kato S, Anderson KL, Anderson SD, Ohama E, Reaume AG, Scott RW, Cleveland DW (1998) Aggregation and motor neuron toxicity of an ALS-linked SOD1 mutant independent from wild-type SOD1. Science 281: 1851–1854 9743498 10.1126/science.281.5384.1851

[bib7] Bruna J, Udina E, Alé A, Vilches JJ, Vynckier A, Monbaliu J, Silverman L, Navarro X (2010) Neurophysiological, histological and immunohistochemical characterization of bortezomib-induced neuropathy in mice. Exp Neurol 223: 599–608 20188093 10.1016/j.expneurol.2010.02.006

[bib8] Cavaletti G, Gilardini A, Canta A, Rigamonti L, Rodriguez-Menendez V, Ceresa C, Marmiroli P, Bossi M, Oggioni N, D’Incalci M, De Coster R (2007) Bortezomib-induced peripheral neurotoxicity: a neurophysiological and pathological study in the rat. Exp Neurol 204: 317–325 17214983 10.1016/j.expneurol.2006.11.010

[bib9] Chaudhry V, Cornblath DR, Polydefkis M, Ferguson A, Borrello I (2008) Characteristics of bortezomib- and thalidomide-induced peripheral neuropathy. J Peripher Nerv Syst 13: 275–282 19192067 10.1111/j.1529-8027.2008.00193.x PMC3741683

[bib10] Corona G, Casetta B, Sandron S, Vaccher E, Toffoli G (2008) Rapid and sensitive analysis of vincristine in human plasma using on-line extraction combined with liquid chromatography/tandem mass spectrometry. Rapid Commun Mass Spectrom 22: 519–525 18228243 10.1002/rcm.3390

[bib11] Csizmadia V, Raczynski A, Csizmadia E, Fedyk ER, Rottman J, Alden CL (2008) Effect of an experimental proteasome inhibitor on the cytoskeleton, cytosolic protein turnover, and induction in the neuronal cells *in vitro*. Neurotoxicology 29: 232–243 18155769 10.1016/j.neuro.2007.11.003

[bib12] Cuervo AM, Dice JF (1996) A receptor for the selective uptake and degradation of proteins by lysosomes. Science 273: 501–503 8662539 10.1126/science.273.5274.501

[bib13] Cuervo AM, Dice JF (2000) Unique properties of lamp2a compared to other lamp2 isoforms. J Cell Sci 113: 4441–4450 11082038 10.1242/jcs.113.24.4441

[bib14] Dictenberg JB, Zimmerman W, Sparks CA, Young A, Vidair C, Zheng Y, Carrington W, Fay FS, Doxsey SJ (1998) Pericentrin and *γ*-tubulin form a protein complex and are organized into a novel lattice at the centrosome. J Cell Biol 141: 163–174 9531556 10.1083/jcb.141.1.163 PMC2132723

[bib15] dos Santos FM, Gonçalves JCS, Caminha R, da Silveira GE, Neves CS, Gram KR, Ferreira CT, Jacqmin P, Noël F (2009) Pharmacokinetic/pharmacodynamic modeling of psychomotor impairment induced by oral clonazepam in healthy volunteers. Ther Drug Monit 31: 566–574 19730280 10.1097/FTD.0b013e3181b1dd76

[bib16] Fortun J, Dunn Jr WA, Joy S, Li J, Notterpek L (2003) Emerging role for autophagy in the removal of aggresomes in Schwann cells. J Neurosci 23: 10672–10680 14627652 10.1523/JNEUROSCI.23-33-10672.2003 PMC6740927

[bib17] Franke WW, Schmid E, Osborn M, Weber K (1978) Different intermediate-sized filaments distinguished by immunofluorescence microscopy. Proc Natl Acad Sci USA 75: 5034–5038 368806 10.1073/pnas.75.10.5034 PMC336257

[bib18] García-Mata R, Bebök Z, Sorscher EJ, Sztul ES (1999) Characterization and dynamics of aggresome formation by a cytosolic GFP-chimera. J Cell Biol 146: 1239–1254 10491388 10.1083/jcb.146.6.1239 PMC2156127

[bib19] Garcia-Mata R, Gao Y-S, Sztul ES (2002) Hassles with taking out the garbage: aggravating aggresomes. Traffic 3: 388–396 12010457 10.1034/j.1600-0854.2002.30602.x

[bib20] Gifondorwa DJ, Robinson MB, Hayes CD, Taylor AR, Prevette DM, Oppenheim RW, Caress J, Milligan CE (2007) Exogenous delivery of heat shock protein 70 increases lifespan in a mouse model of amyotrophic lateral sclerosis. J Neurosci 27: 13173–13180 18045911 10.1523/JNEUROSCI.4057-07.2007 PMC6673412

[bib21] Goetz MP, Toft D, Reid J, Ames M, Stensgard B, Safgren S, Adjei AA, Sloan J, Atherton P, Vasile V, Salazaar S, Adjei A, Croghan G, Erlichman C (2005) Phase I trial of 17-allylamino-17-demethoxygeldanamycin in patients with advanced cancer. J Clin Oncol 23: 1078–1087 15718306 10.1200/JCO.2005.09.119

[bib22] Guo F, Rocha K, Bali P, Pranpat M, Fiskus W, Boyapalle S, Kumaraswamy S, Balasis M, Greedy B, Armitage ESM, Lawrence N, Bhalla K (2005) Abrogation of heat shock protein 70 induction as a strategy to increase antileukemia activity of heat shock protein 90 inhibitor 17-allylamino-demethoxy geldanamycin. Cancer Res 65: 10536–10544 16288046 10.1158/0008-5472.CAN-05-1799

[bib23] Johnston JA, Ward CL, Kopito RR (1998) Aggresomes: a cellular response to misfolded proteins. J Cell Biol 143: 1883–1898 9864362 10.1083/jcb.143.7.1883 PMC2175217

[bib24] Kaushik S, Massey AC, Cuervo AM (2006) Lysosome membrane lipid microdomains: novel regulators of chaperone-mediated autophagy. EMBO J 25: 3921–3933 16917501 10.1038/sj.emboj.7601283 PMC1560360

[bib25] Kawaguchi Y, Kovacs JJ, McLaurin A, Vance JM, Ito A, Yao T-P (2003) The deacetylase HDAC6 regulates aggresome formation and cell viability in response to misfolded protein stress. Cell 115: 727–738 14675537 10.1016/s0092-8674(03)00939-5

[bib26] Kochar DK, Rawat N, Agrawal RP, Vyas A, Beniwal R, Kochar SK, Garg P (2004) Sodium valproate for painful diabetic neuropathy: a randomized double-blind placebo-controlled study. QJM 97: 33–38 14702509 10.1093/qjmed/hch007

[bib27] Kopito RR (2000) Aggresomes, inclusion bodies and protein aggregation. Trends Cell Biol 10: 524–530 11121744 10.1016/s0962-8924(00)01852-3

[bib28] Kumar SK, Rajkumar SV, Dispenzieri A, Lacy MQ, Hayman SR, Buadi FK, Zeldenrust SR, Dingli D, Russell SJ, Lust JA, Greipp PR, Kyle RA, Gertz MA (2008) Improved survival in multiple myeloma and the impact of novel therapies. Blood 111: 2516–2520 17975015 10.1182/blood-2007-10-116129 PMC2254544

[bib29] Kyle RA, Rajkumar SV (2009) Treatment of multiple myeloma: a comprehensive review. Clin Lymphoma Myeloma 9: 278–288 19717377 10.3816/CLM.2009.n.056 PMC3910142

[bib30] Mazumder A, Vesole DH, Jagannath S (2010) Vorinostat plus bortezomib for the treatment of relapsed/refractory multiple myeloma: a case series illustrating utility in clinical practice. Clin Lymphoma Myeloma Leuk 10: 149–151 20371450 10.3816/CLML.2010.n.022

[bib31] Meregalli C, Canta A, Carozzi VA, Chiorazzi A, Oggioni N, Gilardini A, Ceresa C, Avezza F, Crippa L, Marmiroli P, Cavaletti G (2010) Bortezomib-induced painful neuropathy in rats: a behavioral, neurophysiological and pathological study in rats. Eur J Pain 14: 343–350 19695912 10.1016/j.ejpain.2009.07.001

[bib32] Mezey E, Dehejia A, Harta G, Papp MI, Polymeropoulos MH, Brownstein MJ (1998) Alpha synuclein in neurodegenerative disorders: murderer or accomplice? Nat Med 4: 755–757 9662355 10.1038/nm0798-755

[bib33] Mitsiades CS, Hideshima T, Chauhan D, McMillin DW, Klippel S, Laubach JP, Munshi NC, Anderson KC, Richardson PG (2009) Emerging treatments for multiple myeloma: beyond immunomodulatory drugs and bortezomib. Semin Hematol 46: 166–175 19389500 10.1053/j.seminhematol.2009.02.003 PMC2746942

[bib34] Morimoto RI (1998) Regulation of the heat shock transcriptional response: cross talk between a family of heat shock factors, molecular chaperones, and negative regulators. Genes Dev 12: 3788–3796 9869631 10.1101/gad.12.24.3788

[bib35] Munster P, Marchion D, Bicaku E, Lacevic M, Kim J, Centeno B, Daud A, Neuger A, Minton S, Sullivan D (2009) Clinical and biological effects of valproic acid as a histone deacetylase inhibitor on tumor and surrogate tissues: phase I/II trial of valproic acid and epirubicin/FEC. Clin Cancer Res 15: 2488–2496 19318486 10.1158/1078-0432.CCR-08-1930

[bib36] Nawrocki ST, Carew JS, Maclean KH, Courage JF, Huang P, Houghton JA, Cleveland JL, Giles FJ, McConkey DJ (2008) Myc regulates aggresome formation, the induction of Noxa, and apoptosis in response to the combination of bortezomib and SAHA. Blood 112: 2917–2926 18641367 10.1182/blood-2007-12-130823 PMC2556625

[bib37] Ogawa Y, Tobinai K, Ogura M, Ando K, Tsuchiya T, Kobayashi Y, Watanabe T, Maruyama D, Morishima Y, Kagami Y, Taji H, Minami H, Itoh K, Nakata M, Hotta T (2008) Phase I and II pharmacokinetic and pharmacodynamic study of the proteasome inhibitor bortezomib in Japanese patients with relapsed or refractory multiple myeloma. Cancer Sci 99: 140–144 17970782 10.1111/j.1349-7006.2007.00638.x PMC11159153

[bib38] Patel PI, Roa BB, Welcher AA, Schoener-Scott R, Trask BJ, Pentao L, Snipes GJ, Garcia CA, Francke U, Shooter EM, Lupski JR, Suter U (1992) The gene for the peripheral myelin protein PMP-22 is a candidate for Charcot-Marie-Tooth disease type 1A. Nat Genet 1: 159–165 1303228 10.1038/ng0692-159

[bib39] Richardson PG, Badros AZ, Jagannath S, Tarantolo S, Wolf JL, Albitar M, Berman D, Messina M, Anderson KC (2010) Tanespimycin with bortezomib: activity in relapsed/refractory patients with multiple myeloma. Br J Haematol 150: 428–437 20618338 10.1111/j.1365-2141.2010.08264.x PMC3418598

[bib40] Richardson PG, Barlogie B, Berenson J, Singhal S, Jagannath S, Irwin D, Rajkumar SV, Srkalovic G, Alsina M, Alexanian R, Siegel D, Orlowski RZ, Kuter D, Limentani SA, Lee S, Hideshima T, Esseltine D-L, Kauffman M, Adams J, Schenkein DP, Anderson KC (2003) A phase 2 study of bortezomib in relapsed, refractory myeloma. N Engl J Med 348: 2609–2617 12826635 10.1056/NEJMoa030288

[bib41] Richardson PG, Briemberg H, Jagannath S, Wen PY, Barlogie B, Berenson J, Singhal S, Siegel DS, Irwin D, Schuster M, Srkalovic G, Alexanian R, Rajkumar SV, Limentani S, Alsina M, Orlowski RZ, Najarian K, Esseltine D, Anderson KC, Amato AA (2006) Frequency, characteristics, and reversibility of peripheral neuropathy during treatment of advanced multiple myeloma with bortezomib. J Clin Oncol 24: 3113–3120 16754936 10.1200/JCO.2005.04.7779

[bib42] Richardson PG, Sonneveld P, Schuster MW, Stadtmauer EA, Facon T, Harousseau J-L, Ben-Yehuda D, Lonial S, Goldschmidt H, Reece D, Bladé J, Boccadoro M, Cavenagh JD, Boral AL, Esseltine D-L, Wen PY, Amato AA, Anderson KC, San Miguel J (2009a) Reversibility of symptomatic peripheral neuropathy with bortezomib in the phase III APEX trial in relapsed multiple myeloma: impact of a dose-modification guideline. Br J Haematol 144: 895–903 19170677 10.1111/j.1365-2141.2008.07573.x

[bib43] Richardson PG, Xie W, Mitsiades C, Chanan-Khan AA, Lonial S, Hassoun H, Avigan DE, Oaklander AL, Kuter DJ, Wen PY, Kesari S, Briemberg HR, Schlossman RL, Munshi NC, Heffner LT, Doss D, Esseltine D-L, Weller E, Anderson KC, Amato AA (2009b) Single-agent bortezomib in previously untreated multiple myeloma: efficacy, characterization of peripheral neuropathy, and molecular correlations with response and neuropathy. J Clin Oncol 27: 3518–3525 19528374 10.1200/JCO.2008.18.3087 PMC2717758

[bib44] Roodveldt C, Bertoncini CW, Andersson A, van der Goot AT, Hsu S-T, Fernández-Montesinos R, de Jong J, van Ham TJ, Nollen EA, Pozo D, Christodoulou J, Dobson CM (2009) Chaperone proteostasis in Parkinson's disease: stabilization of the Hsp70/*α*-synuclein complex by Hip. EMBO J 28: 3758–3770 19875982 10.1038/emboj.2009.298 PMC2790486

[bib45] San Miguel JF, Schlag R, Khuageva NK, Dimopoulos MA, Shpilberg O, Kropff M, Spicka I, Petrucci MT, Palumbo A, Samoilova OS, Dmoszynska A, Abdulkadyrov KM, Schots R, Jiang B, Mateos M-V, Anderson KC, Esseltine DL, Liu K, Cakana A, van de Velde H, Richardson PG, for the VISTA Trial Investigators (2008) Bortezomib plus melphalan and prednisone for initial treatment of multiple myeloma. N Engl J Med 359: 906–917 18753647 10.1056/NEJMoa0801479

[bib46] Scuteri A, Nicolini G, Miloso M, Bossi M, Cavaletti G, Windebank AJ, Tredici G (2006) Paclitaxel toxicity in post-mitotic dorsal root ganglion (DRG) cells. Anticancer Res 26: 1065–1070 16619507

[bib47] Shen S, Zhang P, Lovchik MA, Li Y, Tang L, Chen Z, Zeng R, Ma D, Yuan J, Yu Q (2009) Cyclodepsipeptide toxin promotes the degradation of Hsp90 client proteins through chaperone-mediated autophagy. J Cell Biol 185: 629–639 19433452 10.1083/jcb.200810183 PMC2711573

[bib48] Wacker JL, Huang S-Y, Steele AD, Aron R, Lotz GP, Nguyen QV, Giorgini F, Roberson ED, Lindquist S, Masliah E, Muchowski PJ (2009) Loss of Hsp70 exacerbates pathogenesis but not levels of fibrillar aggregates in a mouse model of Huntington's disease. J Neurosci 29: 9104–9114 19605647 10.1523/JNEUROSCI.2250-09.2009 PMC2739279

[bib49] Watanabe T, Kato H, Kobayashi Y, Yamasaki S, Morita-Hoshi Y, Yokoyama H, Morishima Y, Ricker JL, Otsuki T, Miyagi-Maesima A, Matsuno Y, Tobinai K (2010) Potential efficacy of the oral histone deacetylase inhibitor vorinostat in a phase I trial in follicular and mantle cell lymphoma. Cancer Sci 101: 196–200 19817748 10.1111/j.1349-7006.2009.01360.x PMC11159849

[bib50] Zou J, Guo Y, Guettouche T, Smith DF, Voellmy R (1998) Repression of heat shock transcription factor HSF1 activation by HSP90 (HSP90 complex) that forms a stress-sensitive complex with HSF1. Cell 94: 471–480 9727490 10.1016/s0092-8674(00)81588-3

